# Research in Vascular Medicine: Where We Are and Where We Are Going

**DOI:** 10.3389/fcvm.2020.00045

**Published:** 2020-03-24

**Authors:** Salah D. Qanadli

**Affiliations:** Cardiothoracic and Vascular Division, Department of Diagnostic and Interventional Radiology, Lausanne University Hospital (CHUV), Lausanne, Switzerland

**Keywords:** vascular medicine, research, innovation, image-guided therapies, wearables devices, endovascular therapies, healthcare

## Introduction

Clinical practice in vascular medicine has greatly changed over the last decades. A close look at the recent past reveals that vascular research has covered an astonishing variety of topics and scopes. Our understanding of vascular diseases is continuously expanding, and new therapeutic approaches are being developed. Multiple factors have driven the evolution of vascular medicine—including disruptive innovations, particularly those involving minimally invasive techniques and technologies; the development of evidence-based practice; and extending the use of minimally invasive interventions beyond traditional vascular interventions. In this commentary, we summarize the main achievements characterizing research in vascular medicine, and future perspectives.

## Where We Are

As our future is largely determined by our past, it is interesting to review the advances and challenges over the last decades. Peripheral arterial disease management has drastically changed, with shifts toward more endovascular services and less open surgery, particularly in arterial diseases. In the USA, peripheral arterial interventions are now more common than coronary interventions, with a significant increase of interventions for distal arteries. Technological advances have played pivotal roles in improving technical success in many settings. Specialized devices have enabled greatly increased rates of crossing chronic total arterial occlusions, even when heavy calcified (1); the long-term safety of vascular access has been improved (2); and outpatient vascular interventions are becoming common at many institutions (3).

Gal et al. (4) recently performed a comprehensive review, and determined that the most important achievements in cardiovascular medicine have been the development of evidence-based concepts to guide treatment, and the establishment of outcomes as a new endpoint for patient management. Analysis of those achievements revealed innovation as the cornerstone for many research areas.

### Modern Evidence-Based Medicine

As minimally invasive techniques grew in popularity, the lack of solid data supporting their use prompted interest in performing clinical trials and meta-analyses to establish stronger evidence in several areas of vascular medicine. For example, one meta-analysis revealed improved outcomes with the use of covered stents in peripheral arterial obstructions compared to bare metal stents (5). The most important and controversial meta-analysis was most likely a 2018 study that reported increased long-term mortality in patients treated with paclitaxel-based drug delivery devices (coated balloons and eluting stents) (6). Since that initial report, several meta-analyses and studies have been conducted to clarify the relationship between paclitaxel and mortality (7, 8). Although some issues remain unclear, these reports highlighted the importance of patient outcome, including long-term follow-up, as a crucial endpoint for decision-making and patient management. Another challenging area of interest was the outcomes of patients with venous thromboembolic disease (9).

### Trends in Innovation and Translational Research

Importantly, the majority of innovations have been disruptive, including techniques and technologies, as well as clinical approaches and unexplored territories. A disruptive innovation, a business model described by Clayton Christensen in the early 1990s, is an innovation that creates a new offer with the potential to disrupt and displace the previously established market. This can include a breakthrough technology that provides better products or that makes products and services affordable and accessible for a larger population. In healthcare, particularly in vascular medicine, disruptive innovations have promoted accelerated development and progression. Thrombectomy devices that assist in crossing chronic total occlusions, including re-entry catheters, have greatly improved technical success rates (10, 11). Drug delivery technologies are among the most important innovations in recent decades (12). Nowadays, endovascular treatment, mainly via percutaneous approaches, can be applied for about 75–95% of vascular lesions (13).

One disadvantage of this rapid technological progress is the difficulty of achieving a high level of evidence, as currently defined in the medical community (14). Multilevel disruptive innovations typically imply frequent changes and rapid improvements of technologies, which limit their evaluation in multiple randomized controlled trials. This is particularly true for new medical devices (15).

## Where We Are Going

Considering the above-described recent history of vascular medicine, a pragmatic research strategy should aim to address current knowledge gaps, develop approaches to help establish future healthcare and human wellness, and promote the culture of innovation. The greatest challenge in addressing the knowledge gap is probably the manner in which knowledge itself is built. The current definition of high-level evidence presents several limitations in the setting of innovative technologies, particularly in “dependent research”—which can be defined as clinical research that depends on a market-available technology provided by a private partner different from those involved in the patient-management decision processes. This recent partnership for sharing knowledge and data science between the academic environment and industry is considered an important driver of innovation and progress. Notably, in such partnerships, short-term objectives (e.g., feasibility, safety, and performances) are more commonly evaluated compared to long-term endpoints and cost-effectiveness. For example, many randomized clinical trials examining drug delivery technologies are performed by industry and, in the post-marketing phases, are usually focused on the short-term and mid-term endpoints required for extending their markets, rather than on long-term performance or safety endpoints. In the near future, it will be necessary to build new models for acquiring independent knowledge and building evidence.

The recent introduction of large data as sources for acquiring new knowledge through deep learning and artificial neural networks will help us meet the challenges of rapidly gathering evidence (16). In fact, these new approaches will affect all medical research and will significantly contribute not only to building evidence but also to redesigning horizons for medical practice. From this perspective, the future of vascular medicine will likely be dominated by preventive medicine, predictive medicine, personalized medicine, and participatory practice. In the near future, predictive medicine will be used to forecast future health events and to predict therapeutic responses for many procedures. For example, recent advances in functional imaging combined with morphologic assessment using computational algorithms might help in predicting the technical success of treatment with regards to endovascular repair of several aortic disorders (17). Personalized medicine is another future development. Although the personalized treatment approach is an old concept—described in ancient Greece by Rufus of Ephesus, who suggested that drugs have different effects on different people—it has attracted increasing interest over recent years.

Clearly, the next decade will bring accelerated research in vascular medicine. In particular, I believe that we will observe progress in the follow four pillars.

### Imaging and Tools for Image-Guided Minimally Invasive Therapies

Imaging should continue to be an important driver for improving patient management—from diagnosis to prediction of disease complications and imaging-guided therapies. Close integration of multimodal imaging, 3D imaging, 3D-printed models, and image fusion may provide better intra-procedural guidance, thus increasing efficiency and precision, especially in complex anatomy. Growing interest in the concept of x-free guidance will drive research to develop realistic platforms and compatible materials for MR-guided interventions and robotics (18, 19). Specific attention should be paid to integrating virtual or enhanced reality in vascular interventions.

### Implanted and Wearable Devices

Multiple wearable devices have been recently introduced in the market—including devices for tracking heart rhythm and daily activity and for hemodynamic monitoring—and their use is rapidly growing. Wearable devices may offer new data regarding patient health (20), which should help monitor patient condition. At the population level, these data serve as part of the larger trend of big data and artificial intelligence, which will likely play a role in better understanding vascular diseases, and defining patients at risk of developing disease or complications. Moreover, the collected data and their analysis will significantly contribute to building new models for establishing evidence.

We will also see continued development of implanted devices and tools that assist with catheter-based therapies. The arena of research covering vascular implantable devices should be dominated by three main directions: miniaturization for improved feasibility and safety, new delivery systems compatible with future imaging guidance (e.g., MRI) or novel implantation concepts (e.g., automation and robotization), and controlling the biologic responses to implanted devices with or without local drug delivery.

### New Endpoints for Patient Management and Healthcare Systems

The current paradigm shift in patient management, from a focus on death to greater focus on patient quality-of-life endpoints, will continue. Additionally, treatment strategies will be increasingly driven by cost-effectiveness. Healthcare systems will shift from a fee-for-service model to a fee-for-value model, with the expansion of systems based on a unified region or country. The trend toward web-based care and telemedicine will present challenges, as we strive to preserve the quality of service and patient security, while improving rapid and affordable service access.

### Education and Training

As rapid innovations prompt rapid changes in practices, new tools, and systems should be developed for education and training to ensure the most efficient possible translation of innovation to clinical practice (21). Finally, great effort should be made to demonstrate the importance of multidisciplinary collaboration for improved patient management, especially in the era of fast-moving technologies.

## Summary and Conclusions

In conclusion, the last decade has brought the development of many new approaches in vascular medicine. However, this rapid advancement significantly limits the ability to build the highest level of evidence to support many recent achievements. Over the next decade, we should endeavor to address knowledge gaps and establish new systems for evidence-based medical practice. This arena will likely continue to be dominated by the disruptive innovations that have characterized vascular medicine to date.

## Author Contributions

SQ designed, drafted, and critically revised the manuscript.

### Conflict of Interest

The author declares that the research was conducted in the absence of any commercial or financial relationships that could be construed as a potential conflict of interest.

## References

[1] Volpi S Chouiter A Saucy F Hajdu S Jouannic AM Qanadli SD. Percutaneous intentional intra-luminal-assisted recanalization (PILAR technique) of challenging chronic total occlusions using a high-frequency vibration device. Eur Radiol. (2018) 28:4792–9. 10.1007/s00330-018-5479-y 29789906

[2] Dwivedi K Regi JM Cleveland TJ Turner D Kusuma D Thomas SM. Long-term evaluation of percutaneous groin access for EVAR. Cardiovasc Inter Rad. (2019) 42:28–33. 10.1007/s00270-018-2072-3 30288590 PMC6267668

[3] Malekzadeh S Rolf T Doenz F Chouiter A Jouannic AM Qanadli SD. Safety of elective percutaneous peripheral revascularization in outpatients: a 10-year single-center experience. Diagn Interv Imaging. (2019) 100:347–52. 10.1016/j.diii.2018.11.009 30573349

[4] Gal D Thijs B Glanzel W Sipido KR. Hot topics and trends in cardiovascular research. Eur Heart J. (2019) 40:2363–74. 10.1093/eurheartj/ehz282 31162536 PMC6642725

[5] Hajibandeh S Hajibandeh S Antoniou SA Torella F Antoniou GA. Covered vs. uncovered stents for aortoiliac and femoropopliteal arterial disease: a systematic review and meta-analysis. J Endovasc Ther. (2016) 23:442–52. 10.1177/1526602816643834 27099281

[6] Katsanos K Spiliopoulos S Kitrou P Krokidis M Karnabatidis D. Risk of death following application of paclitaxel-coated balloons and stents in the femoropopliteal artery of the Leg: a systematic review and meta-analysis of randomized controlled Trials. J Am Heart Assoc. (2018) 7:e011245. 10.1161/JAHA.118.011245 30561254 PMC6405619

[7] Albrecht T Schnorr B Kutschera M Waliszewski MW. Two-year mortality after angioplasty of the femoro-popliteal artery with uncoated balloons and paclitaxel-coated balloons-a pooled analysis of four randomized controlled multicenter trials. Cardiovasc Intervent Radiol. (2019) 42:949–55. 10.1007/s00270-019-02194-w 30843092

[8] Klumb C Lehmann T Aschenbach R Eckardt N Teichgraber U. Benefit and risk from paclitaxel-coated balloon angioplasty for the treatment of femoropopliteal artery disease: a systematic review and meta-analysis of randomised controlled trials. EClinicalMedicine. (2019) 16:42–50. 10.1016/j.eclinm.2019.09.004 31832619 PMC6890981

[9] Tang T Chen L Chen J Mei T Lu Y. Pharmacomechanical thrombectomy versus catheter-directed thrombolysis for iliofemoral deep vein thrombosis: a meta-analysis of clinical trials. Clin Appl Thromb Hemost. (2019) 25:1076029618821190. 10.1177/1076029618821190 30808224 PMC6715002

[10] Bhatt H Janzer S George JC. Crossing techniques and devices in femoropopliteal chronic total occlusion intervention. Cardiovasc Revasc Med. (2017) 18:623–31. 10.1016/j.carrev.2017.06.002 29102343

[11] Wilkins LR Sabri SS. Strategies to approaching lower limb occlusions. Tech Vasc Interv Radiol. (2016) 19:136–44. 10.1053/j.tvir.2016.04.006 27423995

[12] Anantha-Narayanan M Shah SM Jelani QU Garcia S Ionescu C Regan C. Drug-coated balloon versus plain old balloon angioplasty in femoropopliteal disease: an updated meta-analysis of randomized controlled trials. Catheter Cardiovasc Interv. (2019) 94:139–48. 10.1002/ccd.28176 30838719

[13] Veith FJ. A look at the future of vascular surgery. J Vasc Surg. (2016) 64:885–90. 10.1016/j.jvs.2016.07.096 27666442

[14] Fanaroff AC Califf RM Windecker S Smith SC Jr Lopes RD. Levels of evidence supporting American College of Cardiology/American Heart Association and European Society of Cardiology Guidelines, 2008–2018. JAMA. (2019) 321:1069–80. 10.1001/jama.2019.1122 30874755 PMC6439920

[15] Roberts DJ Zygun DA Ball CG Kirkpatrick AW Faris PD James MT. Challenges and potential solutions to the evaluation, monitoring, and regulation of surgical innovations. BMC Surg. (2019) 19:119. 10.1186/s12893-019-0586-5 31455337 PMC6712595

[16] Shameer K Johnson KW Glicksberg BS Dudley JT Sengupta PP. Machine learning in cardiovascular medicine: are we there yet? Heart. (2018) 104:1156–64. 10.1136/heartjnl-2017-311198 29352006

[17] Zhu Y Chen R Juan YH Li H Wang J Yu Z. Clinical validation and assessment of aortic hemodynamics using computational fluid dynamics simulations from computed tomography angiography. Biomed Eng Online. (2018) 17:53. 10.1186/s12938-018-0485-5 29720173 PMC5932836

[18] Wegermann ZK Swaminathan RV Rao SV. Cath lab robotics: paradigm change in interventional cardiology? Curr Cardiol Rep. (2019) 21:119. 10.1007/s11886-019-1218-5 31473815

[19] Heidt T Reiss S Krafft AJ Ozen AC Lottner T Hehrlein C. Real-time magnetic resonance imaging - guided coronary intervention in a porcine model. Sci Rep. (2019) 9:8663. 10.1038/s41598-019-54947-9 31209241 PMC6572773

[20] Pevnick JM Birkeland K Zimmer R Elad Y Kedan I. Wearable technology for cardiology: an update and framework for the future. Trends Cardiovasc Med. (2018) 28:144–50. 10.1016/j.tcm.2017.08.003 28818431 PMC5762264

[21] Andrews C Southworth MK Silva JNA Silva JR. Extended reality in medical practice. Curr Treat Options Cardiovasc Med. (2019) 21:18. 10.1007/s11936-019-0722-7 30929093 PMC6919549

